# Application of the Sinter-HIP Method to Manufacture Cr–Mo–W–V–Co High-Speed Steel via Powder Metallurgy

**DOI:** 10.3390/ma15062300

**Published:** 2022-03-20

**Authors:** Kazuyuki Furuya, Shiro Jitsukawa, Takayuki Saito

**Affiliations:** 1 Department of Industrial System Engineering, National Institute of Technology, Hachinohe College, 16-1 Uwanotai, Tamonoki, Hachinohe, Aomori 039-1192, Japan; saito-c@hachinohe-ct.ac.jp; 2 Former-National Institute of Technology, Fukushima College, 30 Nagao, Hirauearagawa, Iwaki, Fukushima 970-8034, Japan; jitsukawa.shiro@gmail.com

**Keywords:** powder metallurgy, high-speed steel, SKH57, hot isostatic pressing, sintering

## Abstract

1.2C–4Cr–4Mo–10W–3.5V–10Co–Fe high-speed steel (JIS SKH57; ISO HS10-4-3-10) is often manufactured via casting and forging. By applying powder metallurgy, the properties of the abovementioned material can be improved. In this study, the effects of sintering conditions on the formation of precipitates and pores are evaluated. Additionally, strength with and without hydrostatic pressure during sintering is evaluated via static bending and impact tests. Sintering via hot isostatic pressing (HIP) at 1463 K can effectively eliminate pores and prevent the coarsening of precipitates. Toughness and strength improved by 50% by applying HIP.

## 1. Introduction

High-speed steels (hereinafter referred to as HSSs) play an important role in industries because they are widely used, especially in cutting tools [1,2,3,4]. 1.2C–4Cr–4Mo–10W–3.5V–10Co–Fe HSS (JIS SKH57; ISO HS10-4-3-10) is used for cutting tools, as well as for the wear-resistant parts of dies. Carbides containing the alloying elements play an important role to keep hardness at elevated temperatures during cutting [5].

The casting and forging process often results in the coarsening of the carbides to reduce the toughness of SKH57 [6,7]. To improve toughness of the alloy, powder metallurgy (PM) technique is applied. PM is effective for the fine and uniform distribution of the carbides [8,9,10,11,12,13,14].

Sintering with and without isostatic pressing is used. Sintering during isostatic pressure (HIP; hot isostatic pressing) is effective to remove pores in the compacts [15,16,17,18,19]. The metal capsule method is often applied for sintering by HIP [10,20,21]. In this method, metal powder is degassed and encapsulated, and then subjected to HIP.

Often water-atomized powders are used as the raw powders for sintered HSS. The finer the powders, the easier it is to sinter because the specific surface area increases. However, when fine powders are formed in a metal mold, problems with moldability may occur, such as galling due to the fine powder, and poor powder fluidity. Therefore, it was thought that the sintering and moldability could be improved by granulating fine water-atomized powder [22,23].

Moreover, in HIP using the metal capsule method, cost increase is inevitable because of the processes required for capsule fabrication, degassing, encapsuling, capsule removal, and so forth. Therefore, HIP treatment without using capsules was attempted. Normally, in this method, the pores in the compact are closed by other methods before HIP. However, this time, by devising the heat treatment conditions, we decided to close the pores with HIP equipment and continue the HIP treatment in the same equipment. In this way, we aimed to reduce costs by simplifying the process and to accommodate various shapes of products. The quenching and tempering conditions after the HIP process were studied based on the report by Ando et al. [24,25,26]. If the coarsening of precipitates, which leads to a decrease in fracture toughness, can be restricted by the above methods, chipping can be reduced, and it is expected to be applicable to large dies and metal press molds.

In this report, the possibility of improving the properties of SKH57 HSS by applying the PM method is discussed by measuring density, observing microstructure, and conducting hardness, bending, and impact tests on sintered SKH57.

## 2. Materials and Methods

To achieve better performance in HSS manufactured via PM, the size of the raw powder used must be reduced. In this study, raw powder was subjected to water atomization (PF-5F; Epson Atmix Co., Ltd., Hachinohe, Japan), as the powder yielded from this method is finer than that produced via gas atomization [27]. The size of the raw powder was #4000–#3000, with an average particle size of approximately 5 μm. The chemical composition of the raw powder is shown in Table 1.

**Figure 1:**
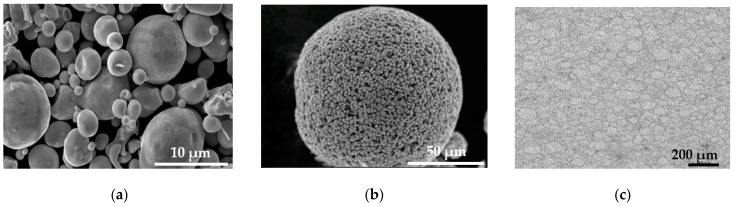
SEM images of water-atomized powders and granulated powder, and OM picture in cross-section of compact. (**a**) Raw powders; (**b**) granulated powder; (**c**) granulated powders after pressing.

The surface of the raw powder was oxidized during water atomization; however, oxygen was released during sintering and was not detected in the sintered material. An SEM image of the raw powders is shown in Figure 1a. As the raw powder particles were extremely fine, only a 10% polyvinyl alcohol (binder) aqueous solution was added to the raw powder to increase the size via spray drying (to prevent the galling of the metal mold). The SEM image of the granulated powder is shown in Figure 1b. The granulated powders were pressed at 600 MPa in the metal mold to form the specimens (compacts). An OM picture in a cross-section of the compact is shown in Figure 1c. The elliptically deformed granulated powders can be clearly seen. The compacts prepared for the bending test measured 36.3 mm long, 10.1 mm wide, and 5.55 mm thick, whereas those prepared for the impact test measured 55.5 mm long, 10.1 mm wide, and 11.1 mm thick.

**Figure 2:**
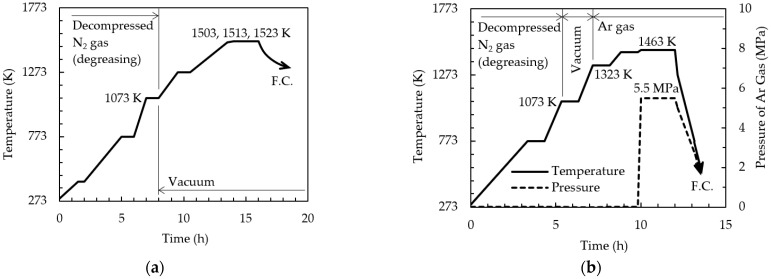
Relationship between time and temperature for sintering (**a**) without S-HIP and (**b**) with S-HIP.

**Figure 3:**
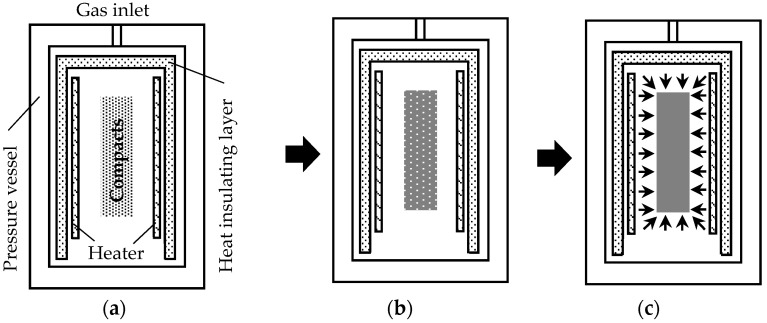
Schematic of HIP furnace and progress of heat treatment. (**a**) Degreasing in decompressed N_2_ gas; (**b**) sintering in vacuum; (**c**) S-HIPping in Ar gas.

Figure 2 shows the relationship between time and temperature for (a) sintering without S-HIP and (b) sintering with S-HIP. Sintering without and with S-HIP were performed using a firing furnace and HIP furnace, respectively. Schematic illustrations of the HIP furnace and the sintering process involving S-HIP are illustrated in Figure 3. In the S-HIP process, the compacts were directly hot isostatically pressed without canning. Typically, the powder is placed in a metal capsule, the inside of the capsule is degassed (canning), and the capsule is hot isostatically pressed with the capsule sealed. In this case, however, by proceeding with sintering in a vacuum prior to pressurization, independent closed pores were formed inside the sample and were not connected to the surface; subsequently, the pores were extinguished via pressurization.

The compacts were heated to 1073 K in a 0.01 Pa N_2_ atmosphere for degassing and removing the binder. Sintering without S-HIP was conducted at temperatures of 1503, 1513, and 1523 K for 2 h in vacuum. Because the sintering temperature often affects the microstructure and mechanical properties, several sintering temperatures were applied. For sintering via S-HIP, heating to 1073 and 1323 K was conducted in a vacuum for degassing the open pores. Subsequently, argon gas was injected into the HIP furnace, and S-HIP was performed at 1463 K for 2 h under a pressure of 5.5 MPa. The sintering temperature with S-HIP was lower than that without S-HIP, which prevented the growth of precipitates and afforded higher strength. After sintering was performed, the compacts were cooled in a furnace.

The sintered bodies were normalized in vacuum at 1133 and 1023 K for 3 and 1 h, respectively, followed by oil quenching after heating in a salt bath at 1513 K for 110–120 s. After quenching was performed, tempering at 833 K for 2 h was performed a few times on the sintered bodies.

Several types of specimens, as shown in Table 2, were prepared. Specimen A was a cast-forged material prepared using the conventional method. Specimens B–D and E were prepared via PM without and with S-HIP, respectively. The immersion density of the specimens was measured, and the results are summarized in the table.

The Rockwell hardness (HRC), bending strength, and Charpy impact value of the specimens were obtained. The hardness was measured with a load of 1471 N and a holding time of 10 s. Meanwhile, the bending strength and impact value were measured using the JIS Z2511 and Z2242 methods for powder metallurgy products, respectively.

**Figure 4:**
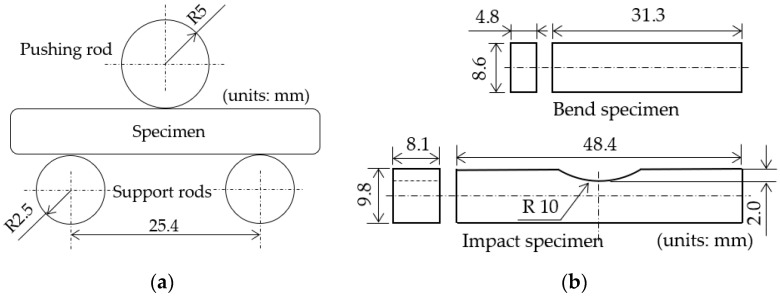
Configuration of jig for bending test and dimensions of bend and Charpy impact specimens. (**a**) Schematic of jig; (**b**) dimensions of specimens.

The configurations of the bend and impact specimens are illustrated in Figure 4. Panel (a) of the figure shows a schematic illustration of the jig for the bending test, and the dimensions of the bend and impact specimens are shown in panel (b). The shape of the bending specimen was rectangular without notches, and the impact specimen had a notch in the center of the specimen, with a depth of 2 mm and a radius of curvature of 10 mm.

## 3. Results and Discussions

### 3.1. Immersion Density and Microstructure

**Figure 5:**
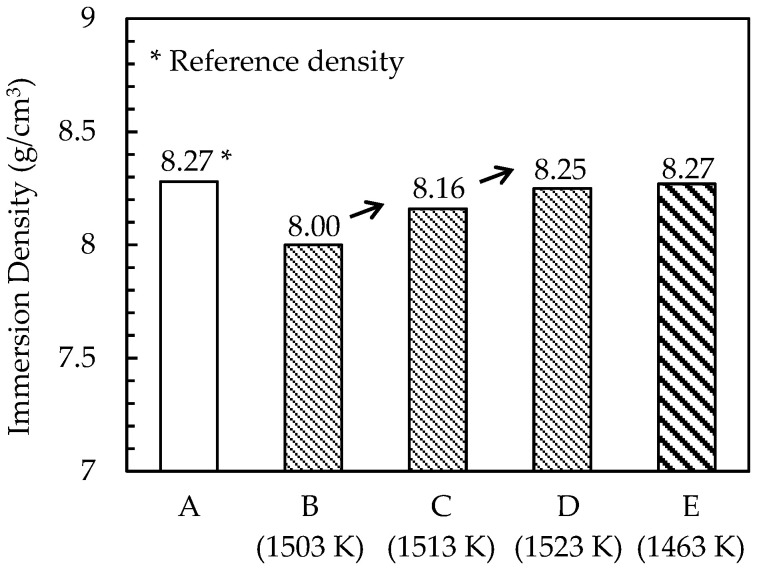
Immersion density change in specimens. Labels A–E represent IDs of each specimen.

**Figure 6:**
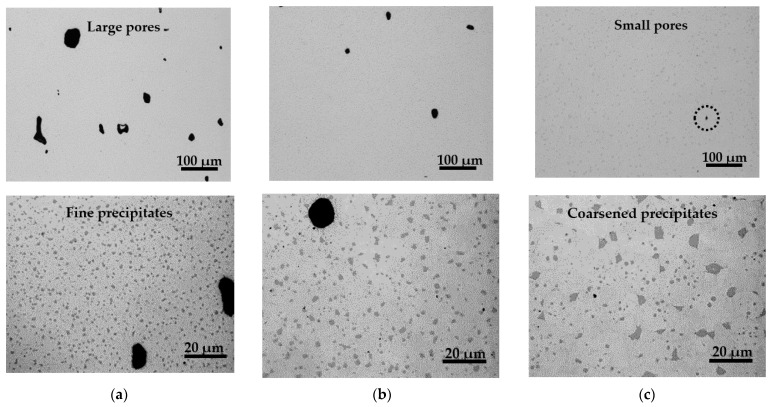
Changes in appearance of pores and precipitates with respect to sintering temperature. Panels (**a**) to (**c**) show that higher sintering temperatures correspond to fewer and smaller pores, but coarser precipitates. (**a**) Specimen B (1503 K); (**b**) specimen C (1513 K); (**c**) specimen D (1523 K).

**Figure 7:**
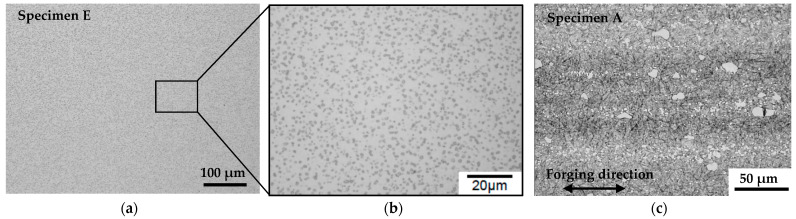
Microstructures of specimens E and A. No pores and fine precipitates were observed in specimen E. In specimen A, large precipitates segregated along forging direction. (**a**) 1463 K sintering with S–HIPping; (**b**) partial magnified view of panel (**a**); (**c**) cast–forged material.

**Figure 8:**
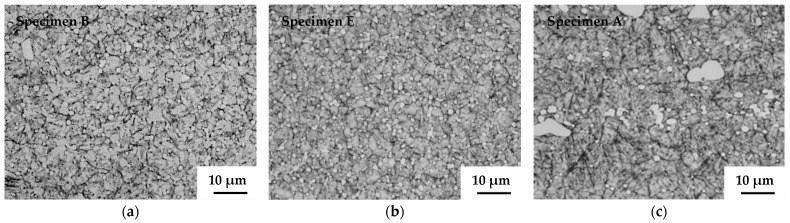
Microstructure of specimens B, E, and A. Size of grains and precipitates between specimens B and E did not differ significantly. In contrast, grains and precipitates of specimen A were larger than those of specimen E. (**a**) 1503 K sintering; (**b**) 1463 K sintering with S–HIPping; (**c**) cast–forged material.

The relationship between immersion density and temperature is shown in Figure 5. Pores and precipitates in the sintered specimens prepared without S-HIP (sintered bodies B, C, and D) were observed under an optical microscope, as shown in Figure 6. Meanwhile, the pores and precipitates in the specimens that underwent S-HIP (sintered body E) and those in the cast-forged material (A) are shown in Figure 7. The precipitates in sintered bodies B (without S-HIP) and E (with S-HIP), and in the cast-forged material (A) are shown in Figure 8.

As shown in Figure 5, the densities of sintered bodies B, C, and D approached that of cast-forged material (A) as the sintering temperature increased. The density of the cast-forged material (A) and sintered–hot isostatically pressed body (E) was 8.27 g/cm^3^. The trend mentioned above is consistent with the volume fraction of pores shown in Figure 6. In addition, almost no pores were detected in the cast-forged material (A) and sintered–hot isostatically pressed body (E), indicating that the S-HIP treatment effectively reduced the number of pores. Figure 6 shows that the precipitate size increased with the sintering temperature. As shown in Figure 6 and Figure 7, the precipitates in the cast-forged material (A) were the largest and occupied the largest volume fraction. Meanwhile, Figure 8 shows that the grain size and precipitate size between sintered body B and the hot isostatically pressed body (E) did not differ significantly.

### 3.2. Mechanical Properties

The cast-forged material (A), sintered bodies B, C, and D, and the sintered–hot isostatically pressed body (E) exhibited similar hardness values of 67–68 HRC. 

**Figure 9:**
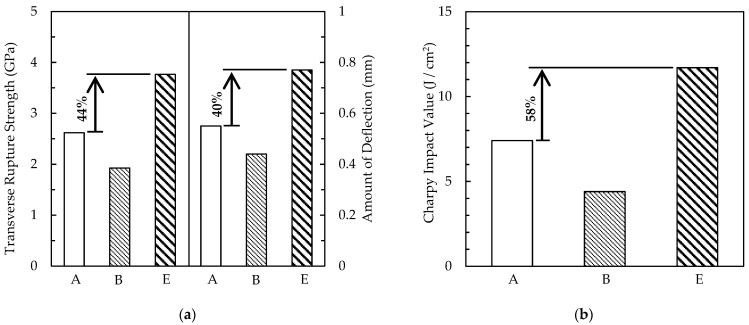
Results of bend and impact tests on specimen A (cast-forged material), B (sintered body), and E (sintered–hot isostatically pressed body). S–HIP significantly improved strength and toughness. (**a**) Comparison of strength and deflection among specimens A, B, and E; (**b**) comparison of toughness among specimens A, B, and E.

Results from the three-point bend tests and impact tests are summarized in Figure 9. The bending strength, deflection, and impact values of the sintered–hot isostatically pressed body (E) increased by 44%, 40%, and 58%, respectively, compared with those of the cast-forged material (A). The sintered–hot isostatically pressed body (E) indicated the highest values for all properties, whereas the properties of sintered body B with large pores were inferior to those of the cast-forged material (A); the pores in sintered body B were larger than 50 μm. These findings suggest that the pores in the sintered body reduced the bending strength, deflection, and impact values. In fact, the stress intensity factor K for a 50-μm-long crack was approximately 15 MPa m^1/2^, which corresponds to the typical value of fracture toughness for cast-forged high-speed steels [28].

The stress intensity factor K (=σ(πa) ^1/2^) was calculated based on a tensile stress of approximately 2000 MPa (measured bending strength of sintered body B) and a crack length of 50 μm (average size of large pores in sintered body B). As failure is dictated by the largest defect in the specimen, and the maximum size of pores in the sintered body is considered to exceed 50 μm, the K value due to pores should be smaller than that for a crack of the same size; however, the calculated K value should correspond to the lower limit.

The bending strength, deflection, and impact values of the cast-forged material (A) were lower than those of the sintered–hot isostatically pressed body (E). Larger precipitates tend to result in a more significant decrease in the strength and toughness [29,30,31], as evidenced by the cast-forged material [29,30,31].

## 4. Summary

The effects of sintering temperature on density, pores, and microstructure were investigated to improve the performance of high-speed steel SKH57 manufactured via powder metallurgy. Meanwhile, the S-HIP method was applied, and the strength and toughness afforded were analyzed. The main conclusions obtained were as follows:

(1) Pores and precipitates in SKH57-sintered HSS depended significantly on the sintering temperature;
(2) S–HIP can sufficiently remove pores even at the lowest sintering temperature (1463 K) in the range investigated in the current experiment;
(3) Significant improvements in terms of strength and toughness were achievable via S–HIP.

## Figures and Tables

**Table 1:** Chemical compositions of SKH57 raw powders (mass%).

	C	Si	Mn	Ni	Cr	Mo	W	V	Co	O	Fe
Measured	1.27	0.34	0.31	0.10	4.28	3.52	9.86	3.45	9.37	0.24	bal.
JIS	1.20–1.35	max0.40	max0.40	max0.25	3.80–4.50	3.00–4.00	9.00–11.00	3.00–3.70	9.00–11.00	--	bal.

**Table 2:** Summary of prepared specimens.

Specimen ID	Method	Sintering Temperature	S-HIP	Immersion Density (g/cm^3^)
A	Casting and Forging	N/A	N/A	8.27
B	PM	1503 K	N/A	8.00
C	PH	1513 K	N/A	8.16
D	PM	1523 K	N/A	8.25
E	PM	1463 K	Applicable	8.27

## Data Availability

The data presented herein are available upon request from the corresponding author.
